# Nanopipettes Enable Native Mass Spectrometry Studies of the Intrinsically Disordered Protein α‑Synuclein in Biochemical Buffers

**DOI:** 10.1021/acs.analchem.6c00544

**Published:** 2026-05-25

**Authors:** Emily J. Byrd, Emma L. Norgate, Joel A. Crossley, Chalmers C. C. Chau, Bob Schiffrin, Alexander Kulak, Sheena E. Radford, Paolo Actis, Antonio N. Calabrese, Frank Sobott

**Affiliations:** † Astbury Centre for Structural Molecular Biology, School of Molecular and Cellular Biology, Faculty of Biological Sciences, 4468 University of Leeds, Leeds LS2 9JT, U.K.; ‡ School of Electronic and Electrical Engineering, University of Leeds, Leeds LS2 9JT, U.K.; § Bragg Centre for Materials Research, University of Leeds, Leeds LS2 9JT, U.K.; ∥ School of Chemistry, University of Leeds, Leeds LS2 9JT, U.K.

## Abstract

Buffer and electrolyte conditions *in vivo* and *in vitro* are known to influence protein structure and function. Intrinsically disordered proteins (IDPs) are particularly sensitive to their solution conditions such as ionic strength and molecular crowding, and their dynamic structural ensembles rapidly respond to the cellular environment. While structural mass spectrometry (MS) techniques are uniquely able to capture aspects of this structural diversity, technical limitations have largely precluded the use of native MS and ion mobility (IM) to interrogate the conformations of IDPs in the presence of different buffers and biologically relevant concentrations of nonvolatile salts. Here, we overcome this challenge by employing sub-100 nm nanopipette electrospray ionization emitters that enable gentle and salt-tolerant analysis to study the conformational distribution of α-Synuclein (αS) monomers using native IM-MS in varied solution conditions, including in phosphate-buffered saline. We show using native MS that it is possible to capture salt- and buffer-induced changes in the αS conformational ensemble in commonly used biochemical buffers, which reflect structural changes from *in silico* predictions and in-solution measurements. This work demonstrates the power of nanopipette emitters for the study of IDPs and establishes native MS as a method that can be routinely used to study the conformational landscape of IDPs in nonvolatile and biologically relevant buffers.

## Graphical abstract

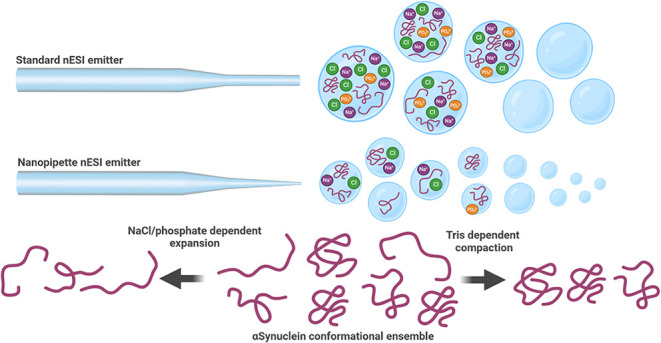

## Introduction

Intrinsically disordered proteins (IDPs) and proteins containing intrinsically disordered regions (IDRs) partake in many protein–protein interactions, aided by their structural plasticity, which facilitates their functional roles in processes such as transcriptional regulation and cell signaling.^1–4^ IDPs are known to explore a vast landscape of rapidly interconverting and coexisting configurations which are determined by their sequence and the chemical composition of their environment.^5^ Many IDPs and IDRs are implicated in disease pathogenesis and are therefore therapeutic targets. This includes diseases such as cancer^6,7^ and neurodegenerative conditions, where many IDPs have been shown to undergo a disorder-to-order transition to form insoluble, β-sheet rich amyloid fibrils.^8–10^ Deposits of amyloid fibrils are associated with a number of neurodegenerative diseases, including Parkinson’s disease (PD), where α-synuclein (αS) fibrils are found in insoluble Lewy body deposits in the substantia nigra of the brain.^11–17^

The structure and function of intrinsically disordered αS monomers are affected by environmental conditions. For example, increasing NaCl concentration (from 50 mM up to 1 M) enhances liquid–liquid phase separation of αS in the presence of crowding agents (PEG400),^18^ by exposing the non amyloid-β component (NAC) domain, a hydrophobic region of the sequence (residues 60–95) through the displacement of water molecules.^19^ This ultimately results in modulation of the aggregation pathway, leading to different fibril morphologies.^20–23^ Moderate physiological concentrations of NaCl (up to ca. 0.1–0.3 M) cause shielding of electrostatic charges between the N-terminal, C-terminal and the NAC domain of αS, resulting in fewer intramolecular contacts and enhancing aggregation into amyloid.^24,25^ Experiments have shown that buffer conditions *in vitro* alter fibril morphology, for example different polymorphs of αS are formed in phosphate and Tris buffers, and at different solution pH values.^26,27^ Additionally, monovalent and divalent ions such as Ca^2+^, Cu^2+^, Cu^+^, Mn^2+^, Mg^2+^, Fe^2+^ and Zn^2+^, many of which are shown to be present at elevated concentration in the brains of Parkinson’s disease patients and are colocalized with αS in Lewy body assemblies,^28,29^ have been identified as regulators of the conformational landscape of αS and as accelerators of amyloid assembly.^28,30–40^ Previous data from hydrogen–deuterium exchange mass spectrometry identified that the conformational behavior of αS differs between solution conditions that match the extracellular, cytosolic and lysosomal environments (which differ in pH and ionic strength), with discrete conformational changes identified in the central hydrophobic NAC domain of αS which forms the amyloid core.^41,42^ These different solution conditions also result in altered αS amyloid assembly kinetics,^41,43,44^ highlighting the importance of replicating physiological conditions when studying IDPs *in vitro*.

In this work, we have analyzed N-terminally acetylated αS, as *in vivo* αS is predominantly acetylated at the N-terminus which has been shown to increase transient N-terminal helical propensity, reduce aggregation rates and alter lipid interactions compared with unacetylated αS.^45–50^ Because acetylation neutralizes the charging of the N-terminal amino group, it may also shift charge-state distributions and modulate long-range electrostatics, which could bias the relative weighting of compact versus extended subpopulations observed by IM-MS. Accordingly, the conformational ensembles discussed here should be interpreted as those of N-terminally acetylated αS, and extrapolation to unacetylated protein should be made with some caution. Nevertheless, the methodological conclusions of this study regarding the use of nanopipettes to reduce nonspecific adduction and improve spectral quality in challenging buffers and matrices apply broadly. Buffer conditions have also been shown to (de)­stabilize protein structures, for example in the case of the yeast prion protein Ure2p, protein stability was increased in Tris-HCl compared to phosphate buffer, and this correlated with an increased lag time for amyloid formation.^51^

Native mass spectrometry (native MS), especially when combined with ion mobility-MS (IM-MS), is able to capture and characterize dynamic, transient, heterogeneous species within a conformational ensemble and is a powerful technique for investigating IDP conformational ensembles.^52–59^ The first step in a native MS experiment is the gentle ionization and transfer of protein species which are kinetically trapped in their solution states into the gas phase. This process is typically achieved using nano electrospray ionization (nESI) under nondenaturing solution conditions so that protein conformations adopted in solution are kinetically trapped and can be measured in the gas phase.^60,61^ The desolvation process that occurs during nESI currently requires the use of aqueous solutions comprising volatile salts such as ammonium acetate (AmAc).^62^ The presence of salts that are commonly required during protein preparation (such as Na^+^, K^+^, PO_4_^3–^ and Tris) as well as buffer additives that mimic the crowded cellular environment and may be critical to maintain the structural integrity of proteins, can cause ion suppression which reduces spectral quality, resolution and sensitivity.^63–66^ Salt ions have also been shown to adduct to protein ions,^67–69^ which can result in unresolvable mass spectra. Therefore, prior to native MS analysis, proteins are typically buffer exchanged from their biochemical buffer (a process which has been simplified recently with online buffer exchange^70,71^ ) into AmAc to dilute/remove salt ions from solution to prevent adduction in mass spectra (ammonium and acetate ions are volatile and do not adduct to proteins). However, many proteins require salts to remain stable in solution or to maintain their native conformational behavior, and produce poorly resolved native mass spectra in AmAc.^69^ One way to circumvent ion adduction is to generate smaller initial droplets during the nESI process by using submicron glass capillary emitters, so that fewer salt ions are partitioned into the solvent droplets containing protein molecules.^72–74^ This approach has been applied successfully to the study of membrane proteins in ionic detergents and globular proteins in nonvolatile biochemical buffers.^68,75^

Using IM to measure the rotationally averaged collision cross section (CCS) distributions of ion species, when coupled to MS is a powerful technique for studying the conformational properties of IDPs in great detail,^37,38,59,76–79^ and we and others have previously used this approach to probe the conformations of αS under different solution conditions (e.g., in the presence of metal ions, dopamine and detergents).^37,38,40,59,80–83^ However, these experiments have been limited to using volatile AmAc based solutions. Alongside submicron nESI emitters, split-barrel theta-capillary emitters have also been shown to enable direct native MS under physiological buffer conditions by exploiting incomplete mixing within a single Taylor cone, with variable mixing times determined by tip diameter and geometry. This generates small salt-depleted droplets that preserve noncolvalent assemblies, but do not represent well-defined buffer compositions.^84–86^ Other approaches rely on in-capillary surface effects for online desalting,^87,88^ but as most salt is removed prior to electrospray samples are not analyzed in the nonvolatile environment.

Here we demonstrate the application of quartz submicron (nanopipette) nESI emitters with a tip diameter of <100 nm (standard borosilicate nESI emitters have diameters of ca. 2–20 μm depending on how they are made; Figure S1 and S2) for native MS of an IDP, using αS in its N-terminally acetylated form as an exemplar.^62,89,90^ While previous work has shown that submicron emitters can be used for nESI and that they enable the acquisition of native mass spectra in diverse buffer conditions,^66,72,74,75,91–93^ these efforts were limited to proof-of-concept studies of stably folded test proteins. Nanopipette emitters have not been previously used to elucidate structural changes involving αS, or conformational changes of other disease relevant IDPs—a class of proteins that are especially responsive to their molecular environment.^76,94,95^ Given the role of solution conditions in tuning the kinetics of αS aggregation^36,41,42,44^ and the experimental evidence demonstrating that different salt (NaCl) concentrations alter its structural properties,^20,24,44^ we chose to use nESI IM-MS to interrogate how ionic strength and buffer composition, known modulators of αS conformation and amyloid propensity,^18–20,44,96^ tune the protein’s conformational distribution. We show that native mass spectra of αS can be acquired in nonvolatile buffers commonly used in biochemical/biophysical studies of protein structure and function *in vitro*, including Dulbecco’s phosphate-buffered saline (PBS), Tris-HCl, potassium phosphate and sodium phosphate, and in the presence of added NaCl. By combining data from IM-MS with *in silico* and in solution measurements of protein conformation, we find that different biochemical buffers have contrasting and apposing effects on the global properties of the ensemble (expansion vs compaction), and that ionic strength tunes the conformational heterogeneity of αS, consistent with recently published in solution measurements^97^ Combined, this work demonstrates that nanopipette nESI emitters can expand the repertoire of possibilities for native MS and IM-MS studies of IDPs, to enable new insights into how IDP conformational ensembles respond to changes in the solution environment and understand how this impacts biological function or in the case of disease, dysfunction.

## Methods

### Protein Expression and Purification

α-Synuclein was produced recombinantly as described previously^38,102^ using competent BL21 DE3 *E. coli* cells that were prepared to stably express the acetyltransferase NatB for N-terminal acetylation of αS. N-terminal acetylation of αS was confirmed by intact denatured liquid chromatography (LC) ESI-MS using a rapid desalting reverse-phase LC precolumn (Figure S3). Five μL of protein at 1 μM concentration in 0.1% (v/v) trifluoroacetic acid was loaded onto an M-class ACQUITY UPLC BEH C4 desalting column (300 Å, 1.7 μm, 2.1 mm × 100 mm, Waters Corporation, Wilmslow, UK). LC separation was performed using solvent A (0.1% (v/v) formic acid in H_2_O) and solvent B (0.1% (v/v) formic acid in acetonitrile) with a gradient of 20–95% B at a flow rate of 50 μL/min. The column was washed with 95% B. The eluate was infused into a Xevo G2-XS Q-Tof MS (Waters, UK) via a Z-spray electrospray source in positive TOF mode with 3 kV capillary voltage, 60 V sampling cone, 80 V source offset, 100 °C source temperature, 250 °C desolvation temperature, 6 V collision energy and a mass range of 350–3000 Da. The instrument was calibrated by a separate injection of 200 pg/μL leucine enkephalin. Hen egg white lysozyme was purchased from Sigma (CAS 12650-88-3).

### Standard nESI Emitter Fabrication

Borosilicate thin wall glass capillaries (0.78 mm) with filament (Harvard Apparatus, UK) were pulled using a P-97 Flaming/Brown micropipette puller (Sutter Instrument, CA, USA) to make two nanospray tips. The optimized procedure used three cycles as follows; heat 560, pull 250, velocity 10, time 50 s. The pulling protocol is specific to the instrument and can vary between different pullers and filaments. Capillaries were placed into a glass Petri dish and coated with palladium in a SC7620 mini sputter coater (Quorum Technologies, Sussex, UK) pressurized with argon at 2 × 10^–2^ mbar. When a current of 25–30 mA was applied the plasma enabled palladium atoms to deposit on the glass surface. The coating time lasted 75 s after which the Petri dish was rotated by 90° followed by a second coating. Samples (10 μL) were loaded into capillaries which were then clipped to size using tweezers.

### Nanopipette nESI Emitter Fabrication

The nanopipette nESI emitter tips were fabricated using 1.0 mm outer diameter and 0.5 mm inner diameter quartz capillaries (QF100-50-7.5; Sutter Instrument) with the SU-P2000 laser puller (World Precision Instruments). A two-line protocol was used: line 1 with HEAT 750/FIL 4/VEL 30/DEL 150/PUL 80, followed by line 2 with HEAT 850/FIL 3/VEL 40/DEL 135/PUL 225. The pulling protocol is specific to the instrument and can vary between different pullers. For native IM-MS measurements, the emitters were loaded with analyte solution (8 μL) and fitted with a platinum wire (PT00-WR-000117; Goodfellow) prior to use.

### Scanning Electron Microscopy (SEM) nESI Emitter Characterization

The pore dimensions of the standard nESI and nanopipette nESI were characterized by field emission SEM with a FEI Nova 450 at an accelerating voltage of 3–5 kV. Images were prepared without coating, using a CBS detector (backscattered electron detector).

### Native Ion Mobility Mass Spectrometry

Native IM-MS experiments were performed on a Waters Synapt HDMS mass spectrometer with traveling (T-wave) ion mobility and a nano-ESI source. N-terminally acetylated αS, expressed and purified as described in ref 38, was analyzed at a concentration of 20 μM in 20 mM AmAc, 20 mM Tris-HCl, 1× PBS (Dulbecco’s A; Thermo Fisher Scientific), 10 mM potassium phosphate, 20 mM sodium phosphate, 100 mM AmAc, and 100 mM Tris-HCl each at a pH of 7.2. For salt titrations, NaCl was added at 12.5 mM, 25 mM and 125 mM. Instrument parameters were set at: capillary voltage 1.0 kV, source temperature 30 °C, backing pressure 0.0–0.3 bar, sampling cone 18 V, extraction cone 1.0 V, trap collision energy 5 V, transfer collision energy 2.0 V, trap DC bias 30 V, IM wave velocity 300 m/s, IM wave height 7.0 V. Gas pressures in the instrument were: trap cell 0.0258 mbar, IM cell 0.36 mbar. ^TW^CCS_N2_ signifies CCS values calculated using traveling wave ion mobility in N_2_ buffer gas using calibrants acquired in N_2_ buffer gas. Lysozyme was analyzed at 10 μM under identical conditions except for: sampling cone 10 V and trap DC bias 12 V. MassLynx V4.1 (Waters Corporation) was used for data processing. The IM spectra were calibrated according to the Bush database^55^ using denatured cytochrome C (charge states 13+ to 19+), myoglobin (charge states 15+ to 24+) and ubiquitin (charge states 7+ to 13+) at 10 μM in 50% (v/v) acetonitrile, 0.1% (v/v) formic acid. Calibrated intensity values were normalized using a scaling factor determined by the signal intensity in the mass spectrum as well as the area under the arrival time distribution peak. Hen egg white lysozyme IM spectra were also calibrated^55^ using native cytochrome C (charge states 6+ and 7+) and myoglobin (charge states 7+ to 9+) measured in 100 mM AmAc. Arrival-time distributions were extracted in MassLynx using fixed *m*/*z* windows around the labeled monomeric charge states. Unless otherwise noted, ATDs were not corrected for potential overlap from oligomer ions (e.g., 8+ monomer overlapping with 16+ dimer). Therefore, reported CCS values represent apparent CCS for the extracted *m*/*z* window. Measurements were taken with *n* = 3 replicates and the standard error was calculated for plotting as shaded areas in the graphs. The apparent radius of gyration (R_app_) was calculated using the following equation:

$$R_{app}=\sqrt{\frac{Average\;CCS}{\pi }}$$

^1^H DOSY NMR

DOSY experiments were performed on a 600 MHz Bruker Avance III spectrometer equipped with a 5 mm QCI-P CryoProbe. For each DOSY experiment under each sample condition, a total of 24 1D acquisitions were performed at different gradient strengths between 2% and 98% where the maximum gradient strength was 48.148 G cm^–1^. To improve data fitting the gradient strength percentages were weighted quadratically toward both 2% and 98% as follows: 2.00, 5.32, 8.76, 12.30, 16.10, 20.00, 24.10, 28.40, 33.00, 38.00, 43.40, 49.40, 50.60, 56.60, 62.00, 67.00, 71.60, 75.90, 80.00, 83.90, 87.70, 91.20, 94.70, 98.00. Following processing, peaks were integrated in the methyl region between 0.6 and 0.9 ppm. To obtain the error for each experiment, the noise at each gradient strength was obtained by integrating a region of the baseline of the same size where no peaks were present (0.3–0.6 ppm). To obtain the diffusion coefficient, following baseline correction and integration, the data were fitted to the following equation:

$$\frac{I}{I_{0}}=e^{-D\gamma^{2}g^{2}\delta^{2}\left(\Delta -\frac{\delta }{3}\right)}$$

where *I* is the observed intensity, *I*_0_ the reference intensity (the signal intensity of the first point), *D* is the diffusion coefficient, γ is the gyromagnetic ratio of the observed nucleus, *g* is the gradient strength, δ the length of the gradient, and Δ the diffusion time. The length of the gradient (δ) and diffusion time (Δ) were 2 and 400 ms, respectively. Baseline adjustment, integration, fitting and plotting was performed with in-house Python3 scripts which made use of the NumPy (v1.26.4),^98^ SciPy (v1.13.1)^99^ and Matplotlib (v3.9.2)^100^ libraries. The integration was performed using the numpy.trapz function. To estimate the uncertainty in the diffusion coefficient a Monte Carlo simulation approach, incorporating the errors associated with each data point, was employed. To simulate the effect of the error in each data point on the diffusion coefficient, we first generated 1000 synthetic data sets. In each data set, every original integrated signal intensity was perturbed by adding random noise drawn from a Gaussian distribution with a mean of zero and a standard deviation equal to the error of that data point. To determine the diffusion coefficient and associated error for each experiment we used the mean and standard deviation of fits to these 1000 data sets. Samples contained 50 μM α-synuclein, 0 mM or 25 mM NaCl, in 100 mM ammonium acetate, pH 7.2, or 100 mM Tris-HCl, pH 7.2, at 24 °C. No viscosity correction was applied, as the change in solvent viscosity between 0 and 25 mM NaCl is much less than 1%,^101^ comparable to the measurement uncertainty in these experiments.

### ThT Amyloid Assembly Kinetics

Kinetics of αS amyloid formation were monitored in a 96-well, nonbinding, flat-bottom, half-area microplate (Corning, USA; 10438082) containing one Teflon polyball (1/8″ diameter; Polysciences Europe) per each well of sample. Samples of 100 μL containing 100 μM αS with 20 μM Thioflavin T (ThT) in 100 mM ammonium acetate, pH 7.2 and 100 mM Tris-HCl with 0 mM NaCl and 25 mM NaCl were incubated at 37 °C shaking at 600 rpm in a FLUOstar Omega plate reader (BMG Labtech). Fluorescence intensity was measured by exciting at 440 ± 10 nm and collecting emission at 482 ± 12 nm using a bandpass filter. Results were blank corrected and normalized to the maximum fluorescence value of each curve.

### Coarse-Grained Simulations

Coarse-grained implicit-solvent simulations of αS were performed in the in the OpenMM framework (version 8.1.1)^103^ using the CALVADOS python package and the CALVADOS2 force field.^104,105^ Unless otherwise stated, simulation parameters used were kept as their default values in the software. The N-terminal positive charge was removed to match the N-terminally acetylated sample used in the MS and NMR experiments. Simulations were performed in a 30 nm^3^ box at pH 7.5. Following energy minimization, simulations were run at a temperature of 25 °C. Simulations were performed for a total of 500 ns each with coordinates being saved every 0.5 ns. The simulation had a time step of 10 fs and a friction coefficient of 10 fs^–1^. The first 5 ns of each simulation was considered to be part of the equilibration time and therefore discarded. In total, 10 simulations were carried out at ionic strengths of 10, 15, 20, 25, 35, 45, 60, 80, 110, and 150 mM. The *R*_g_ was calculated using the inbuilt function in the CALVADOS software for each of the 990 frames outside the equilibration time, with the error bars representing the standard error of the mean.

## Results and Discussion

### Native MS in Physiological Buffers

To evaluate the compatibility of nanopipette emitters with native nESI-MS analysis of αS in biochemical buffers and test their salt tolerance, we used quartz nanopipettes with a pore opening of ca. 40 nm (Figure S1 and S2) to obtain native mass spectra of αS in PBS (Dulbecco’s A). Excitingly, we were able to measure a well-resolved spectrum when using these nanopipette emitters, but (as expected) not when using standard μm-wide emitters (Figure 1a,b). A similar αS charge state distribution was observed with both emitters, suggesting that the smaller tip diameter of the nanopipette does not significantly alter the structural ensemble of monomeric αS, as the charge states adopted in native MS reflects a protein’s solvent exposed surface area.^61,106–109^ While IDP ions can be generated via the chain ejection model (CEM), driven by hydrophobic and electrostatic factors, as well as the charged residue model (CRM), driven by droplet evaporation,^110^ we have found no evidence here for a systematic shift in ionization mechanism or any effects it may have on the observed charge state distributions.

**Figure 1:**
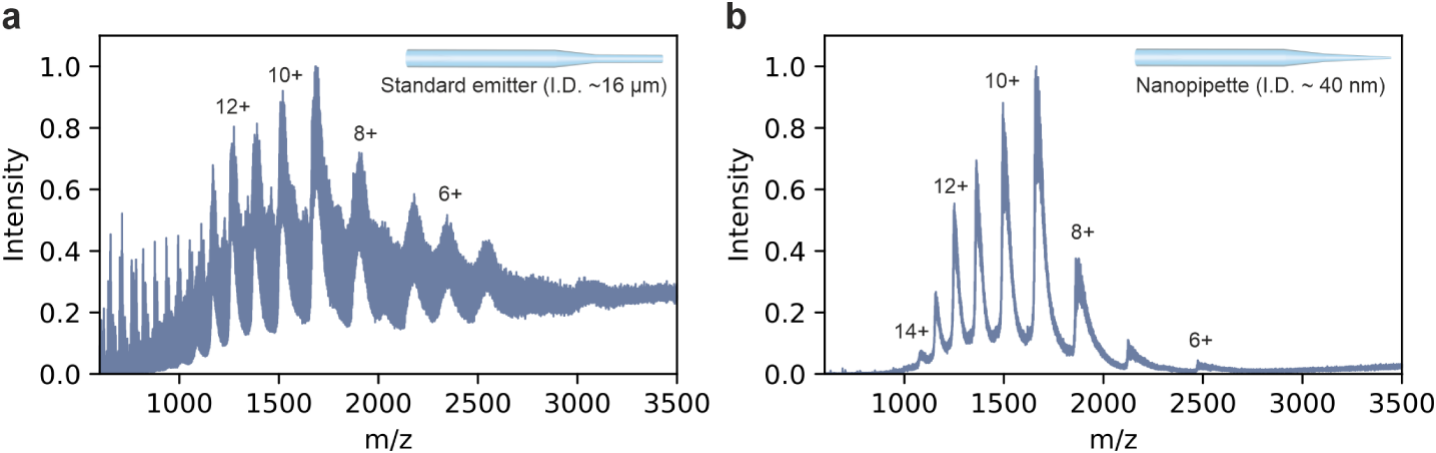
Direct comparison of nanopipette and standard nESI emitters. Representative native nESI mass spectra of 20 μM αS analyzed in PBS, pH 7.2 using (a) standard emitters and (b) nanopipette emitters with identical instrument acquisition parameters on Waters Synapt HDMS (see Methods). I.D.: internal diameter.

In native MS analysis it is common to observe nonspecific adducts and clusters of salts, e.g., Na^+^ (+23 Da) and K^+^ (+39 Da).^111^ Reduced salt clustering and salt adducts are observed with nanopipette emitters (Figure 1b) compared to standard emitters (Figure 1a and Figure S4), consistent with previous reports.^63^ The native mass spectrum of αS in Figure 1b shows an overall decrease in salt adducts, as well as a marked reduction of background salt clusters below 1000 *m*/z. Overall, this shows that nanopipette emitters enable the acquisition of well-resolved nESI mass spectra of αS in biochemical buffers, consistent with previous studies demonstrating their enhanced salt tolerance.^66,112^

When comparing native nESI mass spectra of αS acquired using nanopipettes in PBS (Figure 1b) with those in AmAc buffer (Figure 2a), a shift in the charge state distribution (6+ to 14+ vs 5+ to 18+) occurs as well as the emergence of dimer spectral peaks in AmAc which are absent in phosphate buffers. The most intense charge state in PBS is 9+ whereas the 7+ charge state was most intense in AmAc, indicating a modulating effect of salt on αS conformational behavior, with the higher charge states in PBS indicating conformational extension. Dimers are also observed to varying degrees under some conditions (Figure 2a,c); they are minor species and not pursued further here. Because some dimer charge states overlap in *m*/*z* with monomer charge states (e.g., 8+ monomer with 16+ dimer), the corresponding ATDs may include such contribution. We therefore report CCS values as apparent CCS for the extracted *m*/*z* window and focus interpretation on relative trends across conditions rather than absolute CCS.

**Figure 2:**
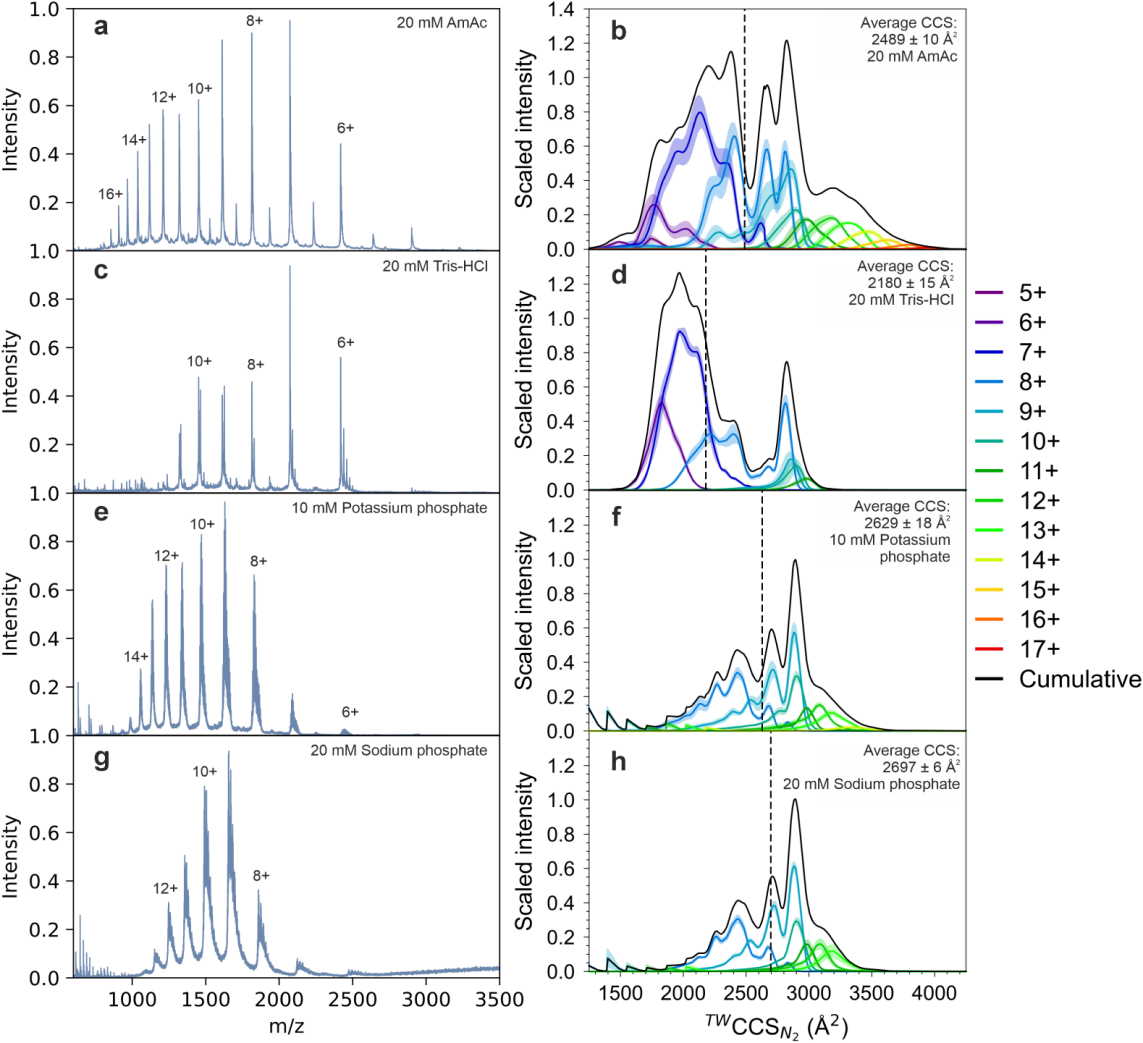
Native MS and IM-MS of αS in biochemical buffers. Native nESI mass spectra of 20 μM αS (left panel) and ^TW^CCS_N2_ distributions (right panel) in (a, b) 20 mM AmAc, (c, d) 20 mM Tris-HCl, (e, f) 10 mM potassium phosphate, (g, h) 20 mM sodium phosphate, each at pH 7.2. Selected charge states are labeled in the mass spectra. The key on the righthand side indicates the color of the CCS distribution for each charge state, the black solid line is the cumulative fit and the black dotted line represents the average ^TW^CCS_N2_ value from the distribution. The shaded region represents the standard error from *n* = 3 replicates.

We next performed native IM-MS to further understand how these changes in the mass spectra are related to structural alterations of αS in a range of commonly used biochemical buffers, by calculating collision cross section (CCS) distributions (see Methods).^55^ Traveling wave (TW) IM measurements were performed in N_2_ buffer gas for each charge state. Consistent with community standards^57^ we refer to these values as ^TW^CCS_N2_. To provide a snapshot of the conformational ensemble captured by the native IM mass spectra, we measured ^TW^CCS_N2_ values for each detected charge state and plotted a cumulative distribution where the contributions from each individual charge state are intensity-weighted and combined (using a scaling factor based on the intensity of each charge state in the corresponding mass spectrum, see Methods). We acquired native mass spectra of αS from the buffers 20 mM Tris-HCl, 10 mM potassium phosphate and 20 mM sodium phosphate, each at pH 7.2 (Figure 2c,e,g), to explore the quality of spectra that could be obtained with the different buffers, and measured IM-MS ^TW^CCS_N2_ distributions from these spectra (Figure 2d,f,h) for comparison with data acquired in conventional AmAc (Figure 2a,b).^113,114^

Using nanopipette emitters, a resolvable charge state distribution was achieved for αS in each of the tested buffer systems, and clear and specific shifts in charge state intensities were observed between buffers (Figure 2a,c,e,g). The native mass spectrum of αS in 20 mM AmAc (Figure 2a) appears as a multimodal distribution of charge states ranging from an extended conformational family (+17 to +10) to a compact conformational family (+9 to +5). This is also reflected in the ion mobility ^TW^CCS_N2_ plot of these data (Figure 2b) which provide much greater detail of the conformational diversity than charge states alone, as shown previously.^38^ To represent the ensembles as a whole and enable a simple comparison between different buffer/salt conditions, we calculated the average (mean) CCS value from the cumulative ^TW^CCS_N2_ distributions (vertical dashed lines in Figure 2). In 20 mM AmAc, this average ^TW^CCS_N2_ value (2489Å^2^) lies in between the extended and compact conformational families (Figure 2b). Interestingly, the spectra and ^TW^CCS_N2_ distributions in PBS (Figure 1b, Figure S5) and the other two phosphate buffers tested (Figure 2e–h) are comparable with each other. All three phosphate-buffered solutions however resulted in ^TW^CCS_N2_ distributions that were narrower and favored more expanded conformations compared to those observed in AmAc (average ^TW^CCS_N2_ values of 2590Å^2^, 2629 Å^2^ and 2697 Å^2^ in PBS, potassium phosphate and sodium phosphate, respectively, compared to 2489 Å^2^ in AmAc). This suggests global conformational effects of phosphate ions on αS, possibly by screening of intramolecular attractive forces between the N- and C-termini. Contrary to this, the spectrum obtained in 20 mM Tris-HCl buffer is shifted to populate lower charge states, and charge state 12+ and above are not observed (Figure 2c). This is reflected in the ^TW^CCS_N2_ distribution which demonstrates that the compact conformational familes are favored in Tris-HCl, with the average value of this ^TW^CCS_N2_ distribution being shifted to a lower value (2180 Å^2^; Figure 2d). It appears that in Tris-HCl, the conformational ensemble is less heterogeneous overall, while maintaining the characteristic two distinct conformational families but less broad than in AmAc. This observation is consistent with previous work that has demonstrated that Tris-HCl has stabilizing effect on the native state of a folded protein compared to phosphate.^51^ Here, we speculate that the Tris-H^+^ ions could coordinate with negatively charged residues such as carboxyl groups, similar to some metal ions which have been found to cause overall compaction.^28,38,102,115,116^ Conversely, the native mass spectra and ^TW^CCS_N2_ distributions of αS in 10 mM potassium phosphate, 20 mM sodium phosphate and PBS are consistent with more expanded αS conformational families than in AmAc, and the CCS fingerprint appears distinctly different to αS in both AmAc and Tris-HCl.

Combined, these results show that the smaller initial droplets enabled by the ∼40 nm diameter orifice of the nanopipettes described in this work enables acquisition of resolvable mass spectra to obtain IM-MS fingerprints of αS in a range of nonvolatile biochemical buffers. The charge state distributions of αS observed in the mass spectra and the ^TW^CCS_N2_ distributions detected using IM-MS both show the same trend, but in opposite directions for Tris-HCl and sodium/potassium phosphate buffers with reference to the volatile AmAc buffer. This suggests that modulation of the conformational landscape of the IDP αS in different buffers can be detected by native IM-MS using nanopipettes, and that such effects are specific to charge shielding and interactions in these solutions (rather than e.g., nonspecific unfolding caused by the high electric fields at the spray tip). Because αS is intrinsically disordered, the conformers observed by IM-MS should not be interpreted as atomistically preserved solution structures. Accordingly, we interpret the observed CCS/arrival-time distributions as reporting relative compactness and shifts in ensemble populations, rather than direct structural replicas of the schematic solution conformers.

### Salt Affects αS Conformational Behavior

Next, we titrated NaCl into AmAc and Tris-HCl buffers to investigate systematically the effect of ionic strength on the conformational families populated by αS using IM-MS (Figure 3). In both AmAc and Tris-HCl, increasing the concentration of NaCl results in αS populating more highly charged species by enhancing the population of more expanded conformational families (Figure S6 and Figure S7, respectively with the 8+ charge state shown in Figure S8). This is supported by analysis of the ^TW^CCS_N2_ distributions which also show a clear shift toward higher average CCS values with increasing salt concentrations, particularly at 125 mM (Figure 3a–h) where an increase in Na^+^ adducts can also be observed (Figure S7). We hypothesize that this shift toward extended conformations is likely due to an overall charge screening effect which disrupts interactions between the negatively charged C-terminal region the overall positively charged N-terminal region,^24^ reducing intraprotein contacts. This is consistent with NaCl-dependent expansion of αS observed previously using small-angle X-ray scattering (SAXS).^19^ The sequence of αS features clusters of the negatively charged residues aspartic acid (D) and glutamic acid (E) within the C-terminal domain (residues 96–140, Figure 3i–j). Since Na^+^ ions present a high affinity for carboxylate side chains it is likely that Na^+^-carboxylate pairing occurs at the C-terminal region of αS.^117^

**Figure 3:**
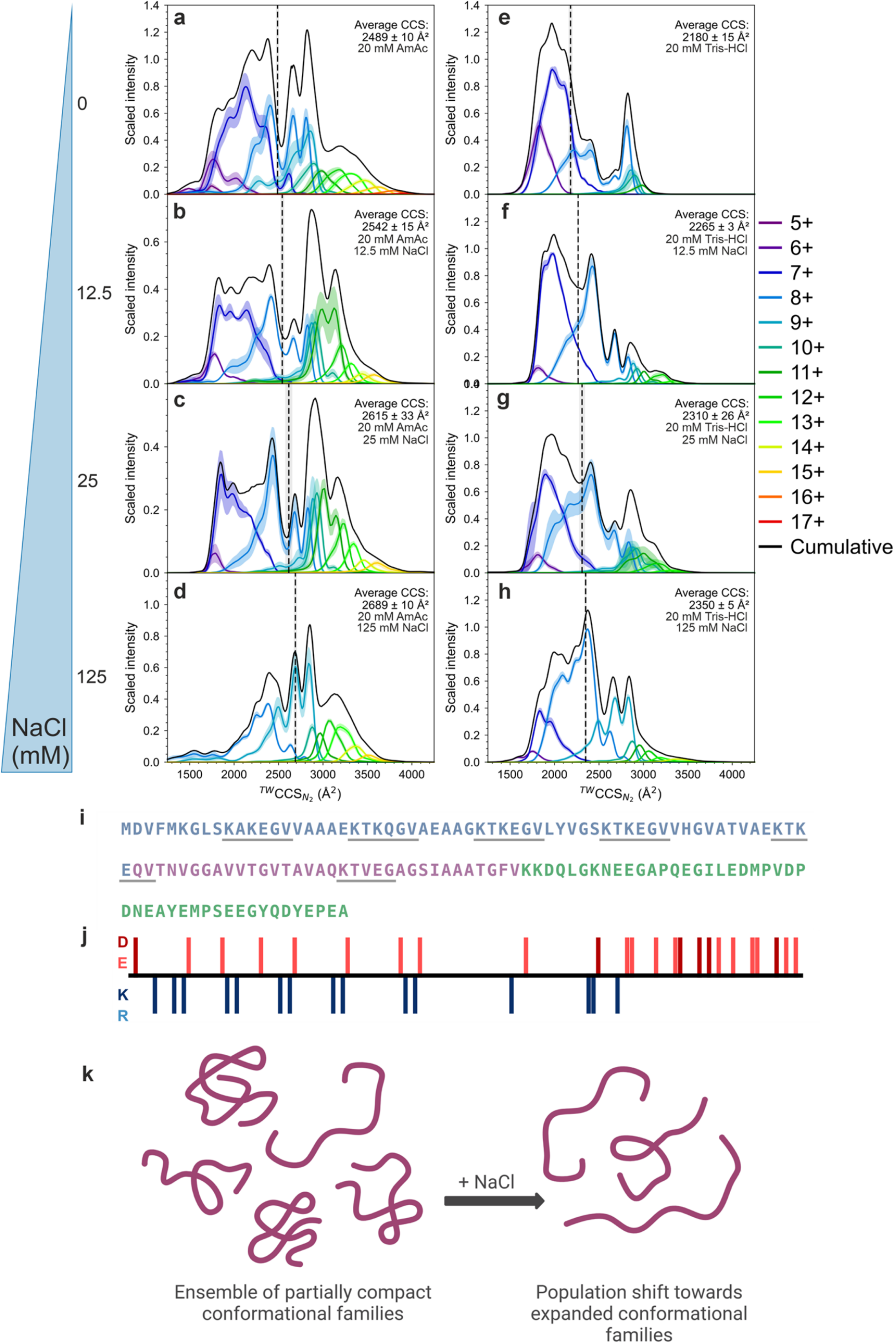
Salt titration of αS in AmAc and Tris-HCl by native IM-MS. ^TW^CCS_N2_ distributions showing the effect of salt (NaCl). αS was measured in (a) 20 mM AmAc with the addition of (b) 12.5 mM, (c) 25 mM and (d) 125 mM NaCl and also in (e) 20 mM Tris-HCl with the addition of (f) 12.5 mM, (g) 25 mM and (h) 125 mM NaCl. The key on the righthand side indicates the color identifying each charge state, the black solid line is the cumulative fit and the black dotted line represents the average ^TW^CCS_N2_ value. The shaded region represents the standard error from *n* = 3 replicates. (i) The sequence of αS which was acetylated at the N-terminus in this study, with residues in blue representing the N-terminal region, in pink the NAC region and in green the C-terminal domain. Imperfect KTKE repeats are underlined. (j) The distribution of charged residues within the αS sequence, negatively charged D and E residues are shown in red and positively charged K residues are shown in blue, there are no R residues within the αS sequence. (k) A schematic of αS expansion in the presence of NaCl.

To determine if the extent of Na^+^ adduction was altering the measured ^TW^CCS_N2_ distributions, we extracted ^TW^CCS_N2_ distributions for two representative charge states (8+ and 10+) with different numbers of Na^+^ bound (up to 12 added Na^+^). Only subtle changes in the ^TW^CCS_N2_ data were detected at different Na^+^ levels with no clear trend (Figure S9), demonstrating that the number of Na^+^ adducts retained/bound to αS in the gas phase is not significantly influencing the conformations measured by IM-MS, but that the changes observed with increasing NaCl levels reflect the global charge shielding effect. As a further control, we compared the effect of adding NaCl on the measured charge states and ^TW^CCS_N2_ distribution of natively folded and disulfide-stabilized hen egg white lysozyme (Figure S10), a well-studied globular protein of similar molecular weight to αS (14 350 Da compared with 14 502 Da for N-terminally acetylated αS used here).^47,49,118^ In both AmAc and Tris-HCl buffers, more compact species and narrower conformational distributions of lysozyme were observed upon addition of 25 mM NaCl, by contrast with the overall extension observed with αS. These opposite trends suggest that the shift in the intrinsically disordered αS ensemble represents specific salt effects in the solution environment which are preserved in the gas-phase and captured with IM-MS, rather than changes in the nESI process induced by the addition of salt. The results are consistent with a model in which the disordered and extended conformational families of αS are stabilized in elevated salt concentrations and that the protein is less driven to form intraprotein contacts which would stabilize more compact states (Figure 3k).

To explore in more detail whether the conformational expansion of αS reflects a general sensitivity to ionic strength, or rather an effect that is specific to adding NaCl, we performed IM-MS of αS at elevated buffer concentrations (100 mM) of volatile AmAc and nonvolatile Tris-HCl buffers. In the case of AmAc, at 20 mM the average CCS of the ^TW^CCS_N2_ distribution was 2489 Å^2^ (Figure 3a) and this increased to 2774 Å^2^ in 100 mM AmAc (Figure 4a). In the presence of an additional 25 mM NaCl we observed a large shift in CCS of αS monomers toward higher, more expanded ^TW^CCS_N2_ values (3171 Å^2^; Figure 4b), with the compact conformational families significantly decreasing in abundance. This is also the case in the 25 mM AmAc/125 mM NaCl buffer (Figure 3d; compact, low-charge states almost abolished), suggesting that the overall ionic strength rather than the specific nature of the ions determines global conformational effects. If the observed effects were instead due specifically to NaCl, then the same 25 mM NaCl concentration in 25 mM AmAc (Figure 3c) and in 100 mM AmAc (Figure 4b) would show comparable CCS effects for αS, which is clearly not the case.

**Figure 4:**
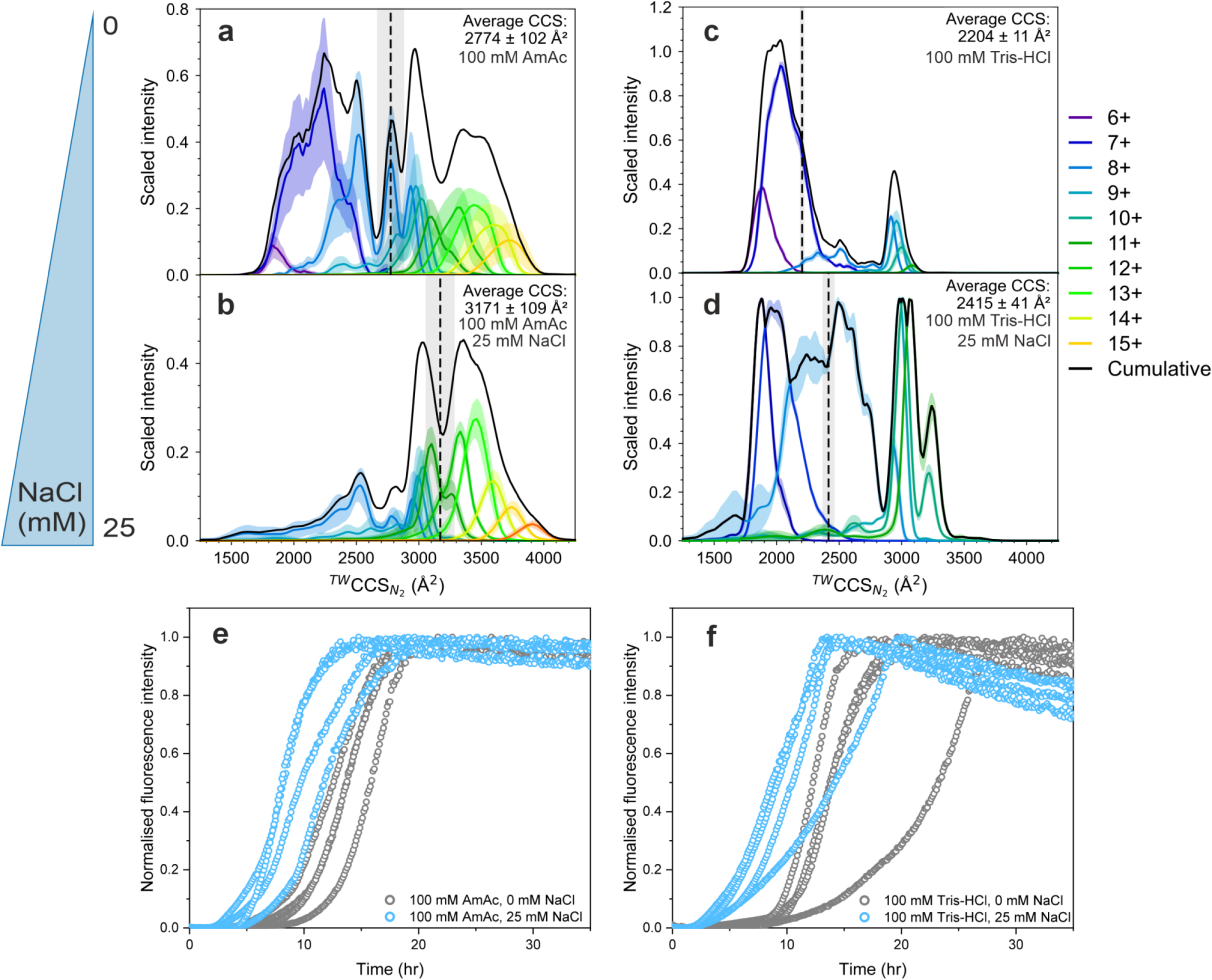
αS undergoes expansion at high ionic strength. ^TW^CCS_N2_ distributions showing the effect of NaCl on αS. αS was measured in (a) 100 mM AmAc with the addition of (b) 25 mM NaCl, and in (c) 100 mM Tris-HCl with the addition of (d) 25 mM NaCl. The key on the righthand side indicates the color of the CCS distribution for each charge state, the black solid line is the cumulative fit and the black dotted line represents the average ^TW^CCS_N2_ value with gray shading indicating the standard error. The shaded region represents the standard error from *n* = 3 replicates. (e) ThT fluorescence in 100 mM AmAc in the absence or presence of 25 mM NaCl, and in (f) 100 mM Tris-HCl in the absence or presence of 25 mM NaCl. Three replicates are shown.

A similar shift toward extended states, but less pronounced, was observed in Tris-HCl buffer upon addition of NaCl (Figure 4c,d; cf. Figure 3e,g), and the combined effect of high NaCl (125 mM) with low Tris-HCl (25 mM) also shows a similar trend (Figure 3h). This is further evidence that the conformational changes that occur in elevated ionic strength conditions are not specific to NaCl, Tris-HCl or AmAc interactions but are instead driven by the overall ionic strength of the solution, most likely via electrostatic shielding. It also agrees well with a recent study which found a strong correlation between monomer conformation and the rate of secondary nucleation of amyloid formation, using kinetic analysis, flow-induced dispersion analysis (FIDA) and coarse-grained molecular dynamics simulations, and demonstrated that this is governed by both global conformation of the polypeptide chain and local contacts in the hydrophobic core domain and acidic C-terminal domain.^97^

Environmental factors including buffer and ionic strength are known to determine amyloid assembly, leading to specific αS fibril morphologies.^119^ Ions can stabilize both intra- and intermolecular hydrophobic interactions through dehydration, promoting fibril assembly.^20,120–122^ Additionally, salt has been proposed to cause expansion of αS, exposing the hydrophobic amyloid-prone NAC domain of the protein leading to accelerated amyloid assembly kinetics.^19,97^ Here, in order to relate IM-MS determined monomeric conformational changes indirectly to amyloid assembly, the kinetics of fibril formation were monitored using Thioflavin T (ThT) fluorescence in the presence or absence of NaCl (25 mM). The results showed that the rate of amyloid assembly increases with the addition of 25 mM NaCl compared to 0 mM NaCl in both AmAc and Tris-HCl buffers (Figure 4e,f).^25,123^ This suggests that the change in structural ensemble induced by added NaCl that we observe with IM-MS impacts the amyloid assembly kinetics of αS, consistent with previous reports.^38,97^ However, the presence of extended states does not always correlate with enhanced aggregation propensity, for example we have demonstrated that Zn^2+^ binding to αS causes compaction and accelerates aggregation.^38,102^ These findings are reconciled by recognizing that Zn^2+^ binding and salt screening act through different mechanisms. Zn^2+^ induces a specific coordination-driven rearrangement, consistent with bridging interactions involving H50 and acidic residues in the C-terminal region (e.g., around D121), thereby stabilizing a distinct subensemble that can favor nucleation. By contrast, increased ionic strength/alkali pairing primarily screens and partially neutralizes the acidic C-terminus, weakening long-range intramolecular contacts that normally suppress aggregation and increasing the availability of aggregation-prone regions. Thus, both “compaction” and “expansion” can accelerate amyloid formation, but via distinct pathways: metal-specific conformational rearrangement versus global electrostatic modulation. More generally, multiple perturbations that reduce effective C-terminal charge (including truncations) are known to increase aggregation propensity, consistent with this electrostatic model.^38,124–126^ This suggests that it will be important to precisely describe the conformational ensembles which are populated under different conditions to understand how they modulate aggregation propensity, moving beyond simple terms such as expansion and compaction. Future work will also be needed to determine whether elevated NaCl or the different buffer systems used here affect the morphology of the fibrils formed.

Because CCS is a global descriptor of a conformational ensemble, an increase in CCS does not uniquely imply a specific local exposure event (e.g., the NAC domain) and can arise from multiple rearrangements within an IDP ensemble. Accordingly, our IM-MS data report a global shift toward more extended conformations under salt screening conditions, but do not directly localize which residues become exposed, or which intramolecular contacts are lost. We therefore interpret the link to faster aggregation as indirect and propose a working model in which partial neutralization/screening of the acidic C-terminus weakens long-range electrostatic contacts (including N–C interactions), thus promoting aggregation-competent conformational states. Testing whether NAC exposure specifically increases under these conditions will require residue-resolved experiments (e.g., HDX-MS, cross-linking MS, NMR)^41,82,127,128^ and/or targeted constructs such as C-terminal truncations or charge-swap variants. Nevertheless, these findings together exemplify that structural rearrangements of αS monomers measured by IM-MS correlate with its aggregation propensity which could help to elucidate disease relevant conformational families for therapeutic intervention.

### Molecular Dynamics Simulations Suggest Expanded States of αS Are Favored at Elevated NaCl Concentrations

Previous coarse-grained molecular dynamics (MD) simulations showed a salt dependent increase in the radius of gyration (*R*_g_) of αS up to a NaCl concentration of 0.1 M as a result of charge shielding.^24^ We sought to expand on these studies using coarse-grained simulations of αS in implicit solvent using CALVADOS.^104,105^ Simulations were carried out at ionic strengths ranging from 10–150 mM (these ionic strengths compare to the total ionic strength of 20 mM AmAc in the absence and presence of between 12.5 and 125 mM NaCl used in IM-MS experiments here) and the *R*_g_ values of the resultant model structures of αS were calculated. These data demonstrate a salt dependent increase in the average *R*_g_ of αS monomers from the simulations over the range of ionic strength values tested (Figure 5a). This change in average *R*_g_ is a result of shifts in the distribution of the overall αS conformational ensemble with increasing ionic strength (Figure S11). We compared these computationally generated *R*_g_ values with the apparent *R*_g_ (R_app_) derived from the weight-averaged mean of the ^TW^CCS_N2_ distributions (see Methods), as a proxy for an ensemble-averaged measurement of CCS (Methods). Similar to the computationally derived *R*_g_ values, we observe an increase in CCS as a function of NaCl concentration in both AmAc and Tris-HCl inferring that αS populates more extended conformations (Figure 5b,c). Examination of representative structures from the simulations at ionic strengths of either 10 mM or 150 mM demonstrates the role of interactions between the N- and C-termini of αS in mediating chain collapse (Figure 5d), consistent with previous reports.^36,129^

**Figure 5:**
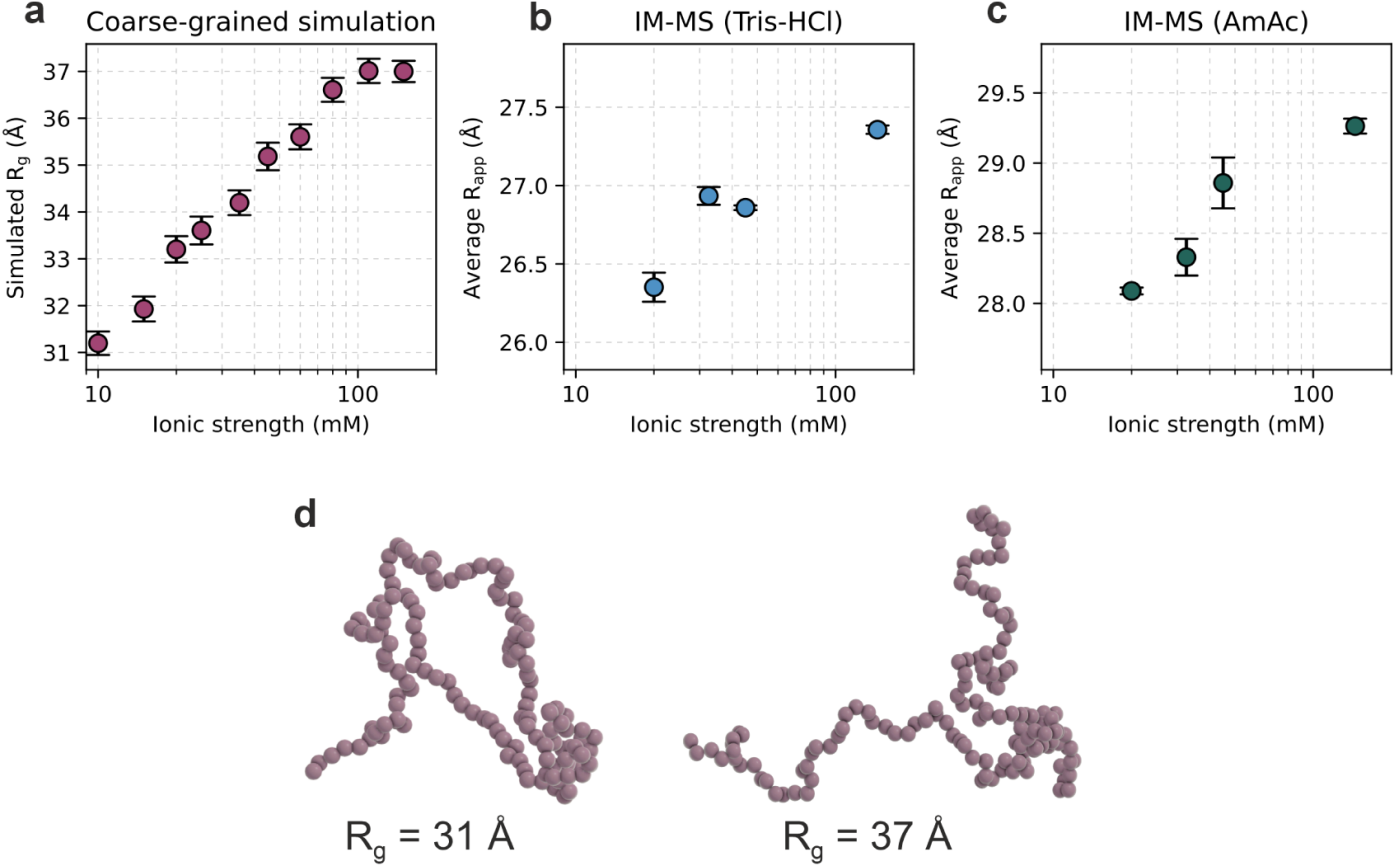
The mean *R*_g_ of αS from coarse grained simulations and average (R_app_) IM-MS values both increase in response to increasing ionic strength. (a) The mean radius of gyration (R_g_) of αS as determined by coarse-grained implicit-solvent simulations at varying ionic strengths (see Methods). The error shown is the standard error of the mean, determined by considering the calculated *R*_g_ values for all αS models in the simulation after a 5 ns equilibration (see Methods for details). (b, c) Average R_app_ calculated from the average ^TW^CCS_N2_ distributions of αS (see Methods, data from Figure 3) in (b) 20 mM Tris-HCl or (c) 20 mM AmAc, with data recorded in the presence of different concentrations of NaCl. (d) Representative coarse grained simulation frames of αS with calculated *R*_g_ values of 31Å and 37Å.

It is important to note that electrospray is a nonequilibrium process. During droplet evaporation and Coulomb fission, ionic strength and droplet composition evolve continuously, and pH and ion pairing may change as the droplet radius reduces. Therefore, a protein cannot be assumed on the ESI timeline to re-equilibrate to a unique ensemble corresponding to a constant ionic concentration. We instead observe kinetically trapped ensembles andthe relationship between solution ensembles and gas-phase IM distributions should be viewed as relative, reproducible shifts in compactness (and population weighting) under different starting solution conditions. For this reason, we use MD simulations at fixed solvent conditions as qualitative support for the direction of ensemble shifts (more/less compact), rather than as a quantitative model of the evaporating droplet.

Next, we sought to compare our ^TW^CCS_N2_ data with solution phase measurements using diffusion-ordered spectroscopy nuclear magnetic resonance (DOSY NMR).^130^ We measured diffusion coefficients for αS in the absence or presence of NaCl (25 mM NaCl) in both AmAc and Tris-HCl buffers (Figure 6a,b,c and Figure S12). The data show that upon addition of NaCl, under both sets of buffer conditions, the measured diffusion coefficient of αS decreases (∼15%). The diffusion coefficient is inversely proportional to the hydrodynamic radius (*R*_h_) of the molecule, suggesting that addition of NaCl results in expansion of αS under the buffer conditions tested.^130,131^ This is consistent with previous small-angle X-ray scattering (SAXS) data that identified a NaCl-dependent increase in the *R*_g_ of αS^19^, and determination of the average *R*_h_ values of αS monomers vs ionic strength determined by Flow Induced Dispersion Analysis (FIDA).^97^ Taken together, these data provide experimental evidence that IM-MS ^TW^CCS_N2_ gas-phase measurements are reflective of the solution-phase conformational distributions of αS.

**Figure 6:**
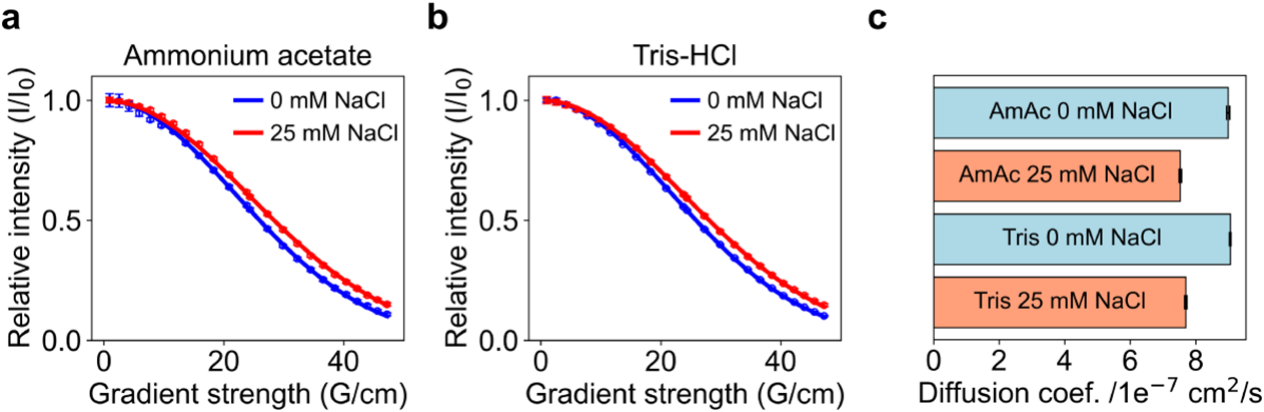
^1^H DOSY NMR indicates expansion of αS upon addition of NaCl. Diffusion coefficients for αS in solution are reduced in the presence of 25 mM NaCl. (a,b) Fitted ^1^H DOSY NMR data for αS in the presence or absence of 25 mM NaCl in (a) AmAc and (b) Tris-HCl buffer. Errors were calculated from the signal-to-noise level in the ^1^H spectra (see Methods). (c) αS diffusion coefficients under different buffer conditions. Errors were calculated using a Monte Carlo simulation approach and the spectral noise (see Methods). Samples contained 50 μM αS, 0 mM or 25 mM NaCl, in 100 mM ammonium acetate, pH 7.2, or 100 mM Tris-HCl, pH 7.2, at 24 °C.

To summarize, we find that R_app_ derived from IM-MS measurements, computationally calculated *R*_g_ values from CALVADOS simulations and diffusion coefficients by DOSY NMR all show a clear expansion/increase in size of αS with increased levels of NaCl (Table S1).

## Conclusions

This study demonstrates that the use of nanopipettes opens the door to IM-MS based measurements of the conformational properties of intrinsically disordered proteins as buffer conditions are altered or counterions are added. The incorporation of nanopipette emitters has enabled us to show using native IM-MS that NaCl and the buffer composition modulate the conformational landscape of αS in specific ways. We show that the conformational expansion of αS in the presence of NaCl (Figure 3), determined using IM-MS, correlates with in solution DOSY NMR measurements and computationally derived *R*_g_ values (Figures 5, 6). Such a change has been reported previously for the folded protein bovine serum albumin (BSA) by analysis of native MS charge state distributions and gas phase thermal denaturation. AmAc was shown to stabilize compact conformational families of BSA and the presence of NaCl shifted the population of BSA toward more unfolded conformations.^112^ IM-MS is well placed to capture snapshots of entire conformational ensembles and provide rich structural information for IDPs, using computational methods to interpret the global changes in conformation observed in molecular detail. Whereas a standard nESI setup mostly limits analysis to volatile buffers which do not represent physiological conditions, nanopipette emitters enable new capabilities for IM-MS through the analysis of samples in common biochemical buffers and high-salt environments.

The ideal nESI emitter geometry is likely dependent on the protein/protein-complexes, biological system and buffers being analyzed, and our comparison of emitters with >10 μm vs sub-100 nm I.D. does not fully capture the full range of emitter regimes. Nevertheless in this work we have benchmarked nanopipette emitters to investigate the effects of biochemical buffers and elevated salt conditions on the conformational ensemble of the IDP αS, and identified an appropriate emitter geometry/diameter to use for this work. We did not find that emitter size and geometry caused protein unfolding, but future work could focus on further emitter optimization, for example by varying I.D. and shape of the tip in order to achieve the best balance between the quality of spectra and ease of use.

While this study focuses on the conformational ensemble of αS monomers, the approach is, in principle, extendable to early oligomeric αS species. Native MS and IM-MS can resolve oligomer stoichiometries and conformers when populations are sufficiently stabilized and abundant;^132–135^ in this context, nanopipette nESI may be advantageous by reducing nonspecific adduction and enabling gentler transfer from higher-ionic-strength buffers, which should help preserve fragile oligomers. Nonetheless, early oligomers are often lowly abundant, and a systematic investigation is beyond the scope of the current study. Further work will determine the molecular weight cutoffs for nanopipette analysis and will expand the amenable analytes which benefit from the improved signal-to-noise offered by nanopipette emitters.

Overall, a clear reduction in αS salt adducts is observed in native nESI mass spectra using the nanopipette emitters descriped here (Figures 1, 2 and Figure S4) which we attribute to the finer droplet spray.^136,137^ The smaller droplets that emerge from the nanopipette partition the solvent into smaller volumes that contain fewer salt ions on average which reduces adduct formation with proteins observed with standard ESI emitters. The application of nanopipette emitters opens the door to analysis of proteins in buffers closer to their native cellular environment, and to deploying native IM-MS to afford a new understanding of how the conformations of IDPs relate to their function or dysfunction, such as the role of αS in synucleopathies. Our approach also paves the way for the structural analysis of other IDPs in complex solution environments which mimic biologically relevant conditions (cellular electrolytes, salts, crowding and liquid–liquid phase separation promoting agents), and demonstrates that there is no need to avoid nonvolatile salts in native IM-MS of IDPs. Future studies exploring the addition of other buffer components, and the introduction of complex sample environments such as extracts from cellular compartments and cell lysates, provide additional avenues to advance this methodology further.

## Supplementary Material



## Notes

Fabrication and characterization of nanopipette ESI emitters; MS of N-terminally acetylated αS; native MS and ^TW^CCS_N2_ distribution of α-Synuclein in PBS buffer; native MS of αS in AmAc and Tris-HCl with NaCl; native MS and ^TW^CCS_N2_ distribution of lysozyme; *R*_g_ values from MD simulations; DOSY NMR of αS; comparison of αS expansion at 20 mM and 125 mM ionic strength (PDF)
