# Supplementary material for: Nanopipettes Enable Native Mass Spectrometry Studies of the Intrinsically Disordered Protein α‑Synuclein in Biochemical Buffers
Source: Anal Chem. 2026 May 25;98(22):16219–33. doi: 10.1021/acs.analchem.6c00544 (PMC13261622; doi:10.1021/acs.analchem.6c00544)
Supplement: Supplementary file 1 [file ac6c00544_si_001.pdf]

Supplementary information

Nanopipettes Enable Native Mass Spectrometry Studies of the Intrinsically Disordered Protein α-Synuclein in Biochemical Buffers

Emily J. Byrd[1]•, Emma L. Norgate[1]•, Joel A. Crossley[1], Chalmers C. C. Chau[2,3], Bob Schiffrin[1], Alexander Kulak[4], Sheena E. Radford[1], Paolo Actis[2,3*], Antonio N. Calabrese[1*], Frank Sobott[1*]

[1]Astbury Centre for Structural Molecular Biology, School of Molecular and Cellular Biology, Faculty of Biological Sciences, University of Leeds, Leeds, LS2 9JT, UK.

[2]School of Electronic and Electrical Engineering, University of Leeds, LS2 9JT, UK.

[3]Bragg Centre for Materials Research, University of Leeds, LS2 9JT, UK.

[4]School of Chemistry, University of Leeds, LS2 9JT, UK.

• These authors contributed equally to this work.

*Correspondence to: f.sobott@leeds.ac.uk, a.calabrese@leeds.ac.uk, p.actis@leeds.ac.uk

Contents

Figure S1. Fabrication of the nESI emitter tips. S3

Figure S2. Comparison of nanopipette emitters with standard nESI emitters. S3

Figure S3. Deconvolved mass spectrum of denatured N-terminally acetylated αS. S4

Figure S4. Zoomed in native nESI mass spectra of the 8+ charge state of αS. S5

Figure S5. Representative $^{TW}CCS_{N2}$ distribution of αS in PBS buffer using a nanopipette nESI emitter. .... S**Error! Bookmark not defined.**

Figure S6. Native nESI mass spectra of αS in 20 mM AmAc with NaCl titration. S6

Figure S7. Native nESI mass spectra of αS in 20 mM Tris-HCl with NaCl titration. S6

Figure S8. Native nESI mass spectra of the 8+ charge state........ S6

Figure S9. Different Na^{+} : αS binding stoichiometries result in some variation of measured CCS distributions but no clear trend. S7

Figure S10. Charge state distribution and $^{TW}CCS_{N2}$ Ion mobility distributions of lysozyme using nanopipette nESI emitters. S8

Figure S11. Distributions of calculated Rg values for αS from coarse grained molecular dynamics simulations. S8

Figure S12. Example 1D spectra of αS from DOSY NMR experiments........ S9

Table S1. Table comparing measurements of αS expansion at 20 mM and 125 mM ionic strength........ S9

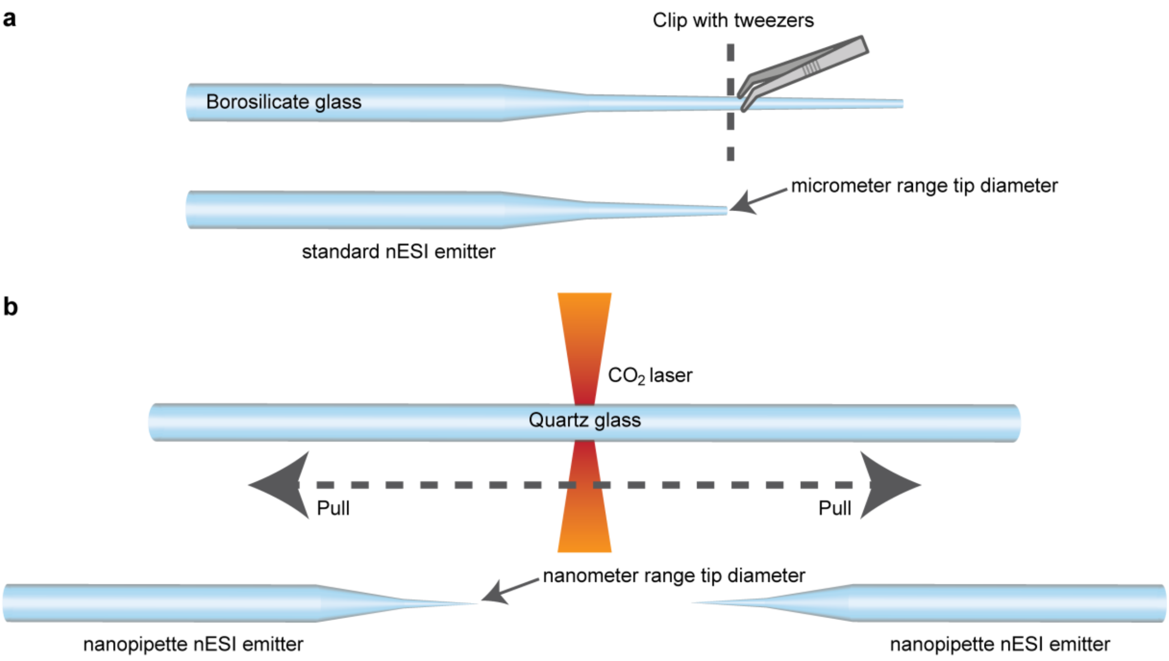

Figure S1. Fabrication of the nESI emitter tips. *The typical fabrication process for the standard nESI emitter tip (a) starts with a pre-pulled borosilicate glass pipette with a long taper. The long taper is clipped with tweezers, resulting in a tip diameter in the 2 - 20 micrometre range. For the fabrication of the nanopipette nESI emitter tip (b), a laser puller is used with quartz glass capillaries. This process results in the fabrication of a pair of nearly identical nanopipette nESI emitter tips, each with a nanometre range sized pore.*

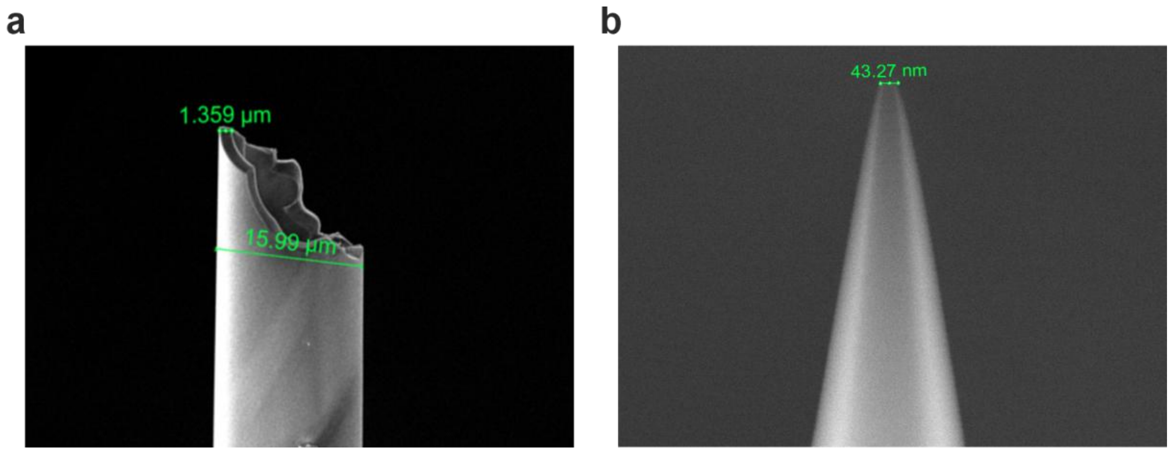

Figure S2. Comparison of nanopipette emitters with standard nESI emitters. *SEM images of (a) standard borosilicate nESI emitters clipped to size with tweezers (pore diameter ~ 16 µm) and (b) of nanopipette emitters (pore diameter ~ 43 nm).*

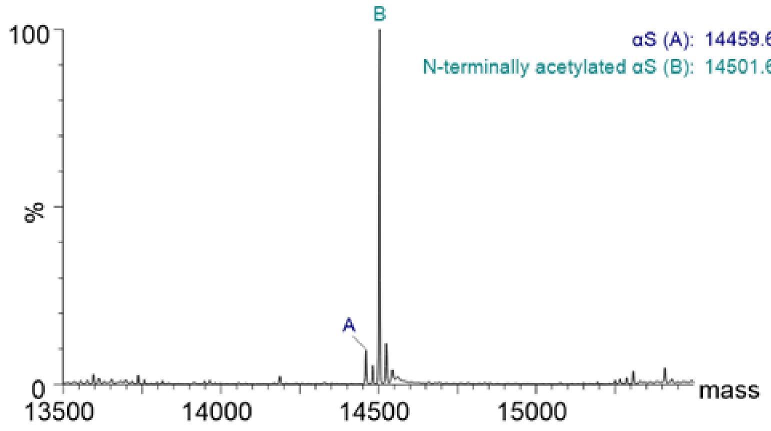

***Figure S3. Deconvolved mass spectrum of denatured N-terminally acetylated αS.** A = αS and B = N-terminally acetylated αS. Protein was measured at a concentration of 1 μM in 0.1% (v/v) trifluoroacetic acid on a Xevo G2-XS QToF instrument (Waters Corporation, Wilmslow, UK).*

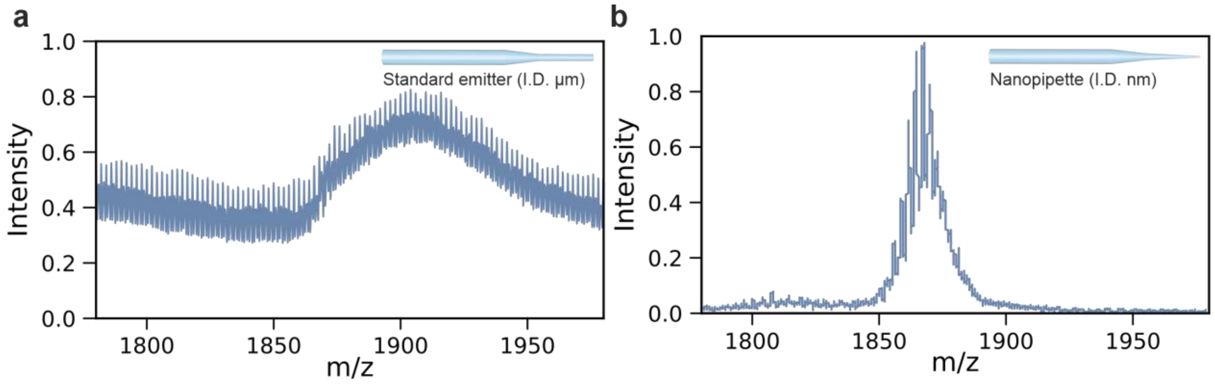

***Figure S4. Zoomed in native nESI mass spectra of the 8+ charge state of αS.** Measured in PBS, pH 7.2 using (a) standard borosilicate emitters and (b) quartz nanopipettes. The predicted m/z of the apo 8+ αS charge state is 1813. The full mass spectra are shown in Figure 1.*

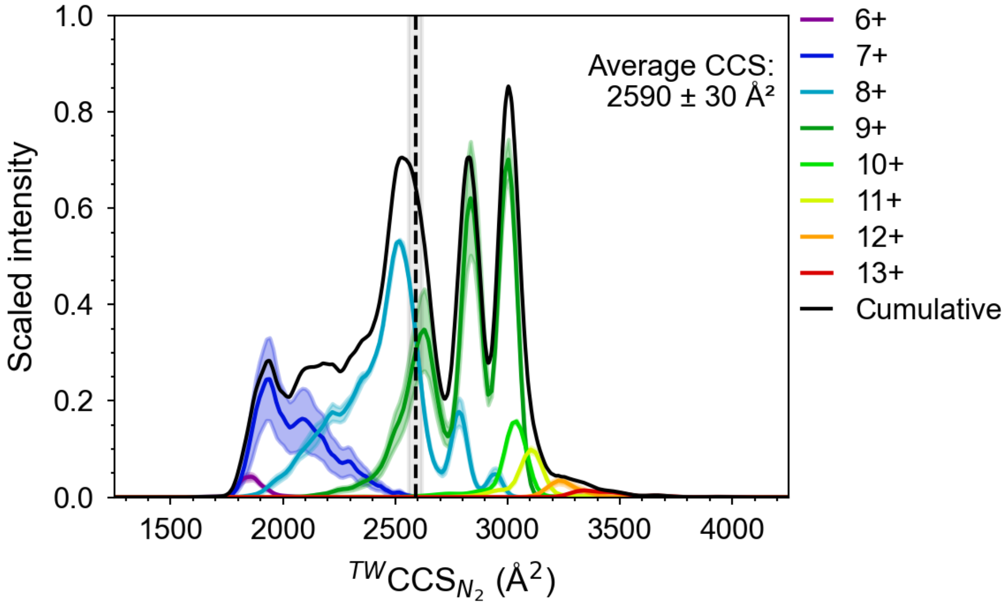

Figure S5. Representative $^{TW}CCS_{N2}$ distribution of αS in PBS buffer using a nanopipette nESI emitter. *The data are taken from a spectrum acquired of 20 µM αS in PBS buffer, pH 7.2. The key on the righthand side indicates the colour of the CCS distribution for each charge state, the black solid line is the cumulative fit and the black dotted line represents the average $^{TW}CCS_{N2}$ value. The shaded region represents the standard error from n = 3 replicates.*

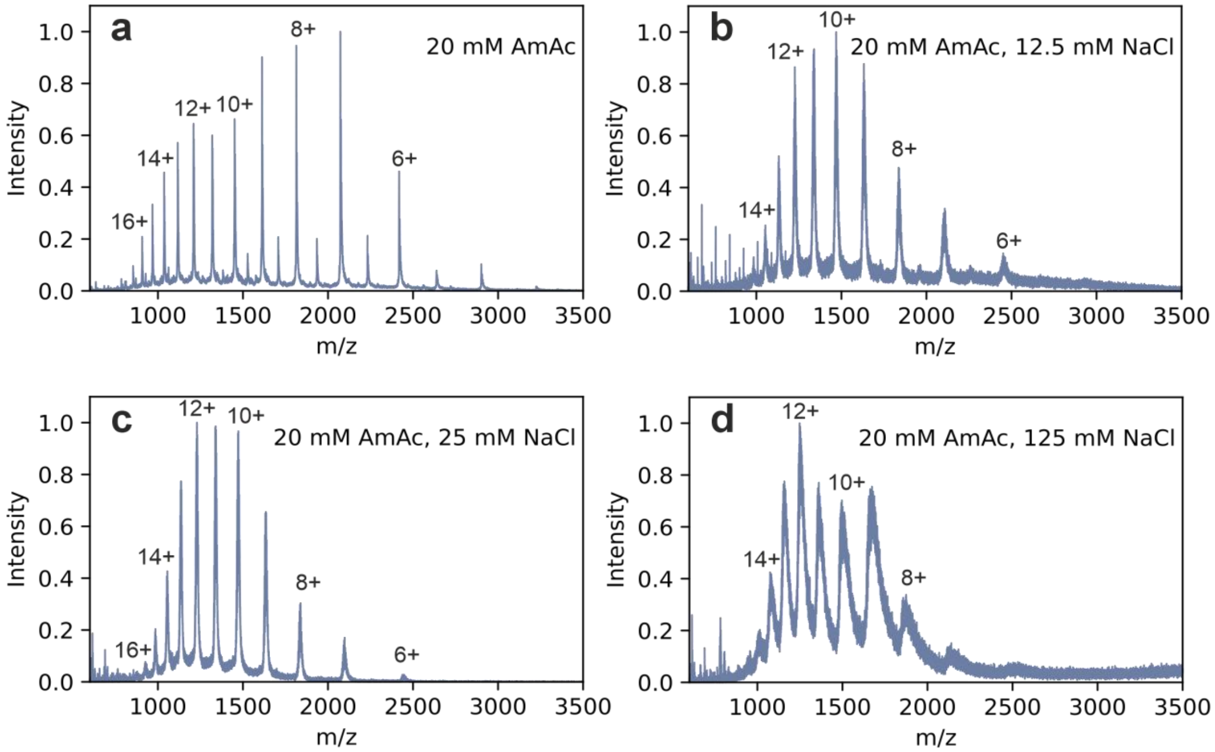

Figure S6. Native nESI mass spectra of αS in 20 mM AmAc with NaCl titration. *αS (20 µM) was measured in (a) 20 mM AmAc, pH 7.2 with the addition of (b) 12.5 mM NaCl, (c) 25 mM NaCl, (d) 125 mM NaCl. Corresponding ion mobility spectra are shown in Figure 3a-d.*

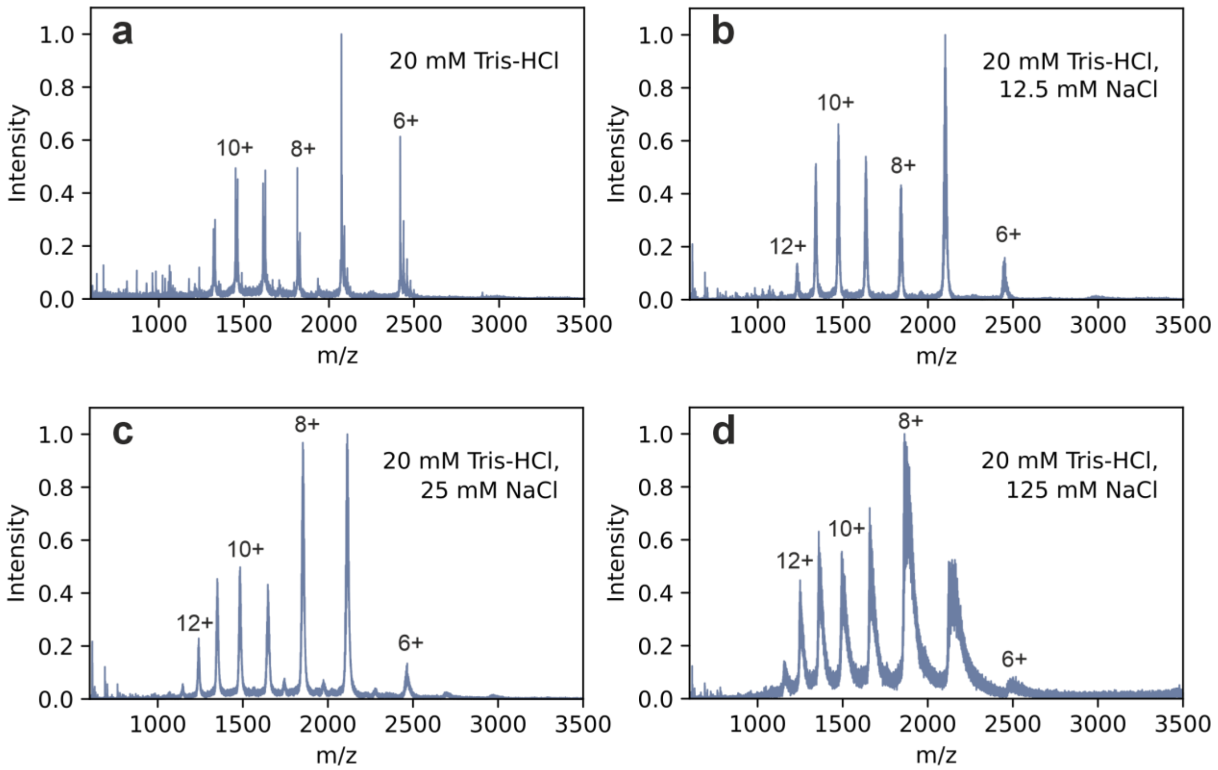

Figure S7. Native nESI mass spectra of αS in 20 mM Tris-HCl with NaCl titration. *αS (20 µM) was measured in (a) 20 mM Tris-HCl, pH 7.2 with the addition of (b) 12.5 mM NaCl, (c) 25 mM NaCl, (d) 125 mM NaCl. Corresponding ion mobility spectra are shown in Figure 3e-g.*

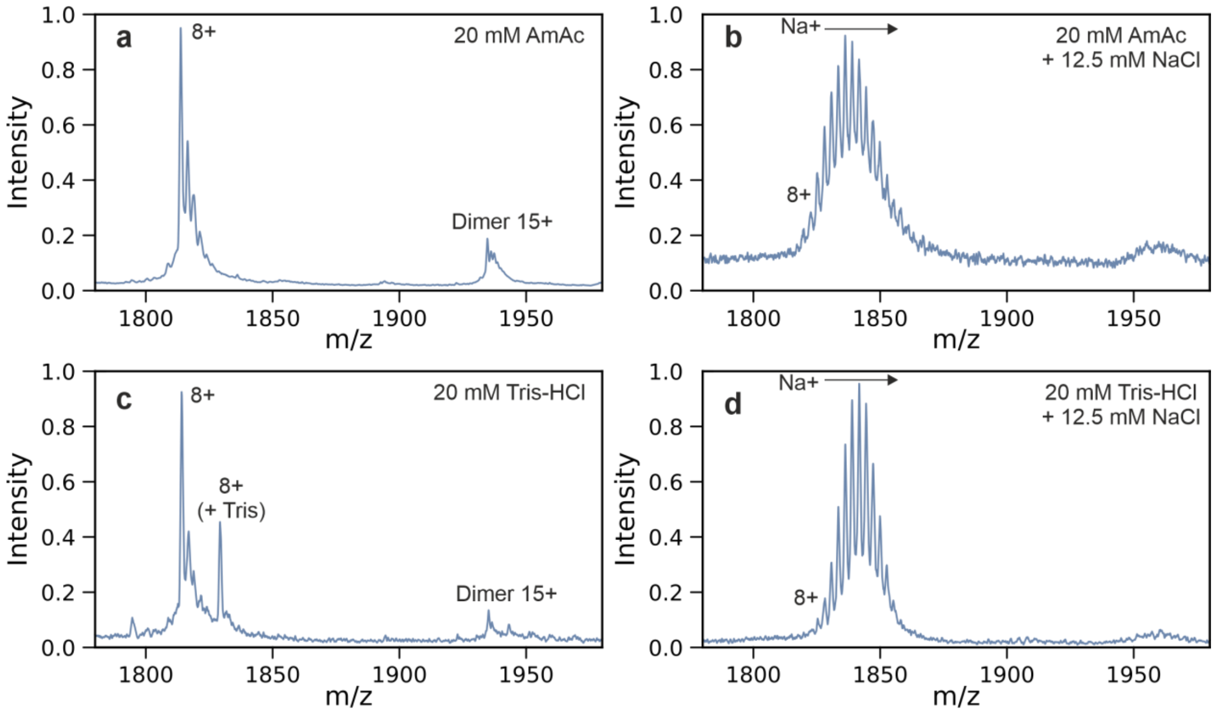

Figure S8. Native nESI mass spectra of the 8+ charge state. *αS measured in (a) 20 mM AmAc, (b) 20 mM AmAc with 12.5 mM NaCl, (c) 20 mM Tris-HCl and (d) 20 mM Tris-HCl with 12.5 mM NaCl. All measurements were taken at pH 7.2. Corresponding full mass spectra and ion mobility spectra are shown in Figure S6a,b, Figure S7a,b, and Figure 3a,b,e,f, respectively.*

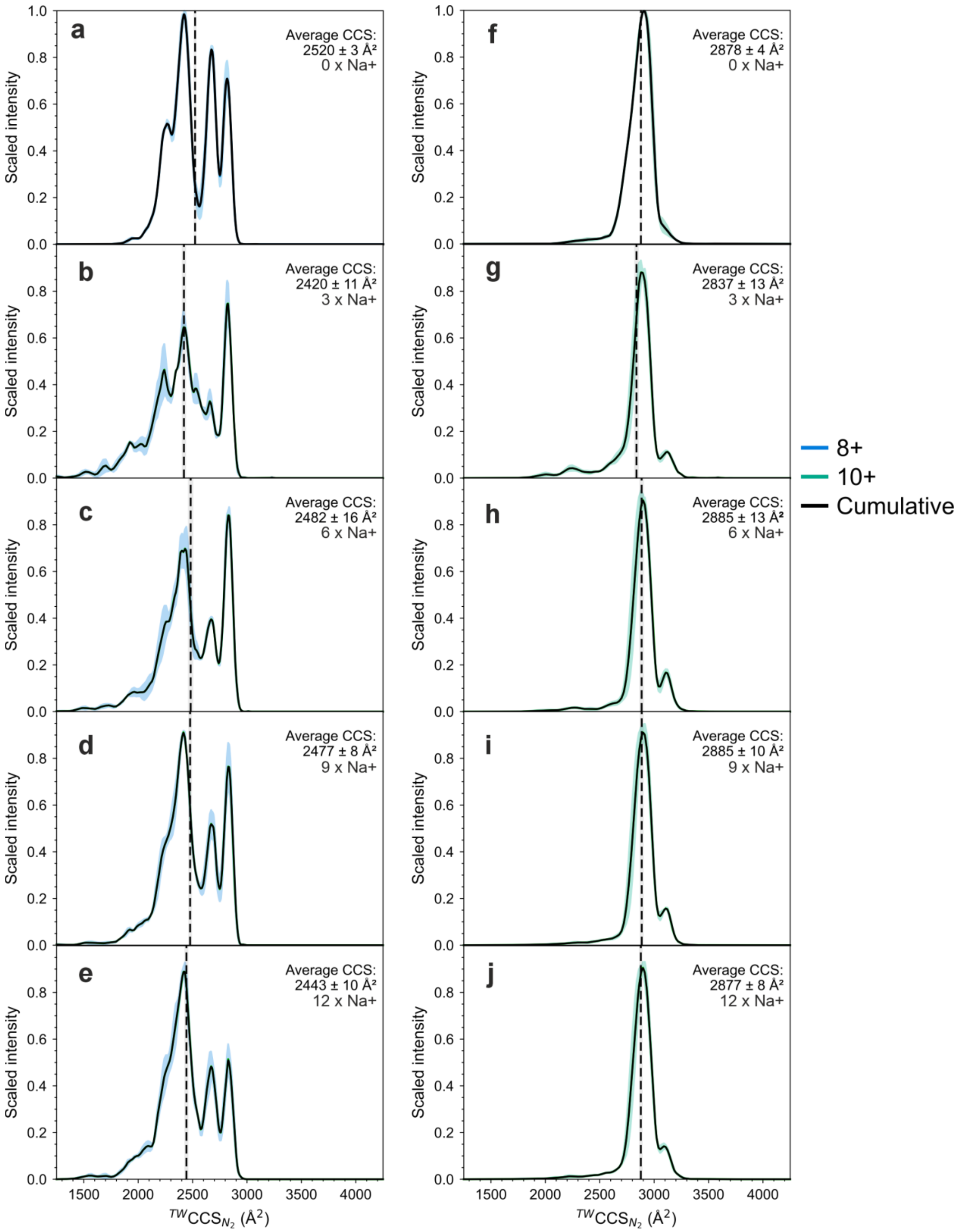

Figure S9. Different Na$^+$: αS binding stoichiometries result in some variation of measured CCS distributions but no clear trend. *Native nESI mass spectra acquired for N-terminally acetylated αS in 20 mM AmAc with 25 mM NaCl (cf Figure 3c). Calculated $^{TW}CCS_{N2}$ values for the 8+ charge state are shown in panels (a)-(e) where (a) is from the spectral peak corresponding to 0 Na$^+$ bound, (b) 3 x Na$^+$, (c) 6 x Na$^+$, (d) 9 x Na$^+$ and (e) 12 x Na$^+$. Calculated $^{TW}CCS_{N2}$ values for the 10+ charge state are shown in panels (f)-(j) where (f) corresponds to 0 Na$^+$ bound, (g) 3 x Na$^+$, (h) 6 x Na$^+$, (i) 9 x Na$^+$ and (j) 12 x Na$^+$. The shaded region represents the standard error from n = 3 replicates.*

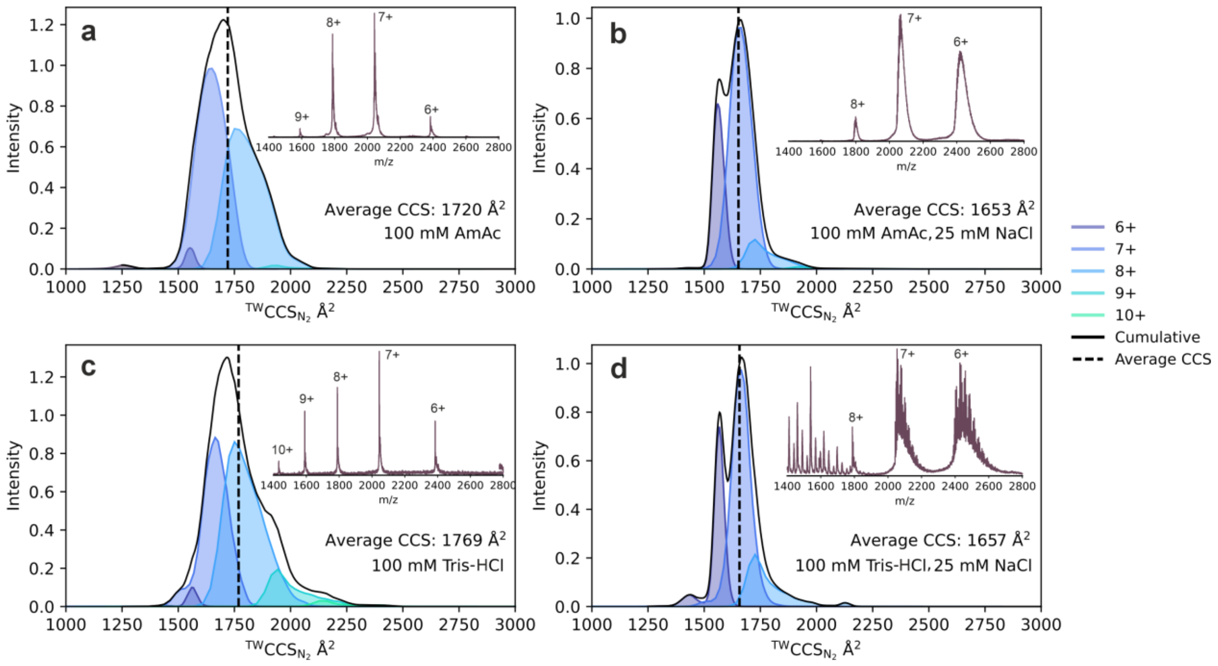

Figure S10. Charge state distribution and $^{TW}CCS_{N2}$ Ion mobility distributions of lysozyme using nanopipette nESI emitters. *nESI mass spectra were acquired of 10 µM hen egg white lysozyme in (a) 100 mM AmAc (pH 7.2), (b) 100 mM AmAc with 25 mM NaCl, (c) 100 mM Tris-HCl and (d) 100 mM Tris-HCl with 25 mM NaCl.*

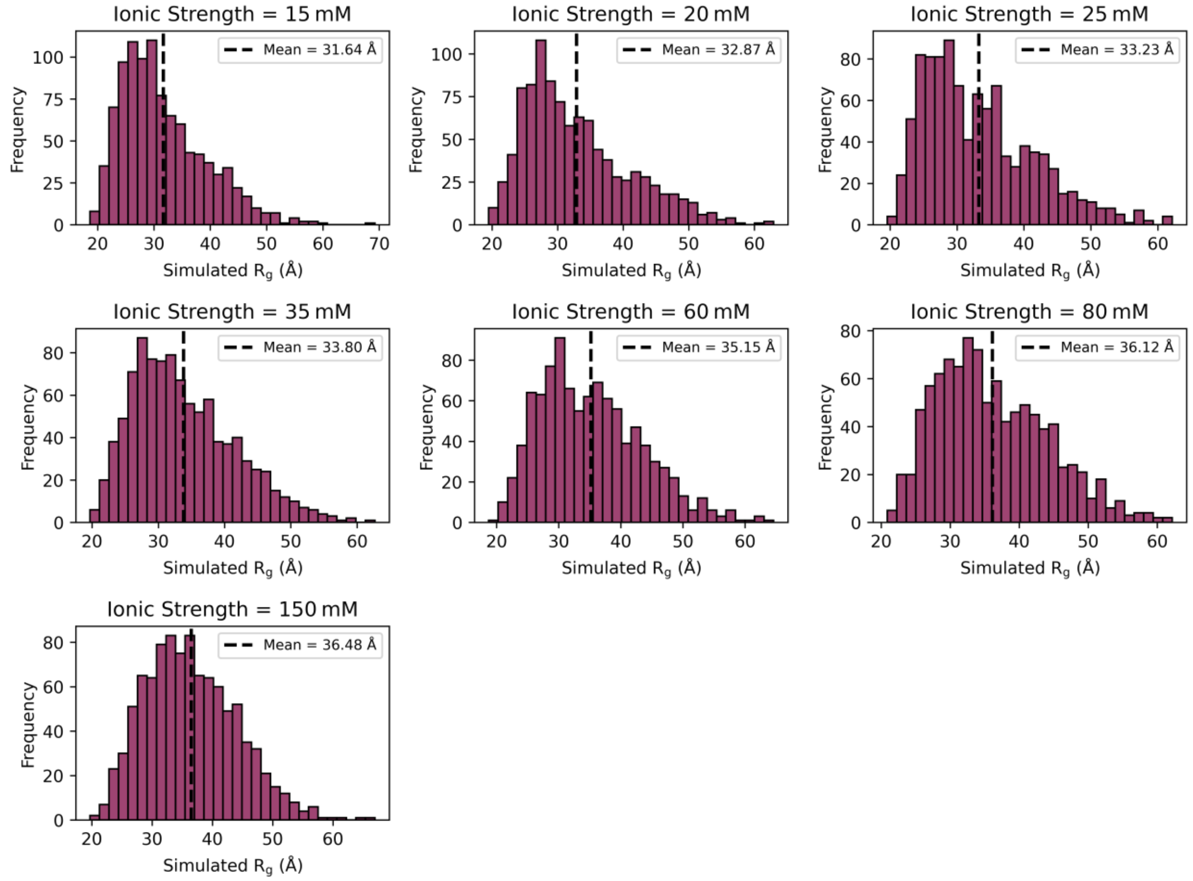

Figure S11. Distributions of calculated R_g values for αS from coarse grained MD simulations. *Each panel shows a different ionic strength value; the mean R_g is shown as a vertical black dashed line. For details on the simulation and number of frames used, see Methods.*

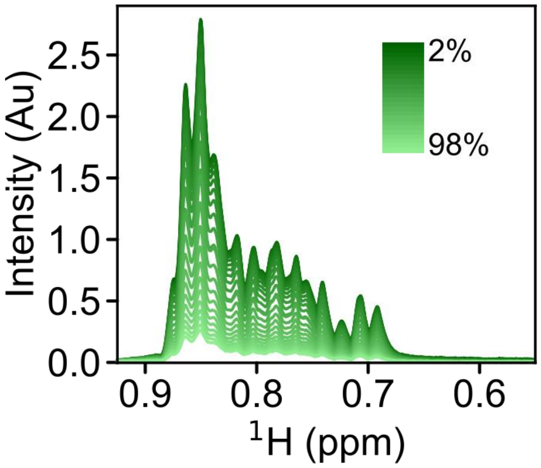

Figure S12. Example 1D spectra of αS from DOSY NMR experiments. *For each experiment under each sample condition, a total of 24 1D acquisitions were made at different gradient strengths between 2% and 98%, where the maximum gradient strength was 48.148 G cm^{-1}. Data for the region used for integration (0.6-0.9 ppm) are shown. The sample contained 50 μM αS in 100 mM Tris-HCl, pH 7.2, at 24˚ C.*

**Ionic Strength**	**R_{app} (Å)**	**% change**	**R_g**	**% change**	**Diffusion coefficient × 10^{-7} (cm^2 s^{-1})**	**% change**
20 mM	28.14	+8%	32.87	+11%	8.99 ± 0.05	-16.2%
125 mM	30.27		36.48		7.53 ± 0.04	

Table S1. Table comparing measurements of αS expansion at 20 mM and 125 mM ionic strength. *R_{app} in Å was calculated from the average $^{TW}CCS_{N2}$ values for αS in 20 mM AmAc, pH 7.2 and 100 mM AmAc with 25 mM NaCl, pH 7.2 (see methods for calculation). R_g was calculated from coarse grained CALVADOS simulations of αS at 20 mM and 125 mM ionic strength, pH 7.5. Diffusion coefficient (cm^2/s) were measured using DOSY NMR.*
